# Vascular surgery core curriculum for medical degrees in Brazil: a proposal

**DOI:** 10.1590/1677-5449.202400392

**Published:** 2026-02-09

**Authors:** Alexandre Yoshiharu Shiomi, Juliano Mendes de Souza, Diancarlos Pereira de Andrade, Marcos Arêas Marques

**Affiliations:** 1 Clínica Angioprime, Curitiba, PR, Brasil.; 2 Universidade Federal do Paraná – UFPR, Curitiba, PR, Brasil.; 3 Faculdades Pequeno Príncipe, Curitiba, PR, Brasil.; 4 Universidade do Estado do Rio de Janeiro – UERJ, Hospital Universitário Pedro Ernesto, Rio de Janeiro, RJ, Brasil.

**Keywords:** curriculum, competency-based education, medical education, vascular diseases, vascular surgical procedures, internship and residency

## Abstract

**Background:**

Vascular surgery (VS) is the medical area focused on the study of the vascular system, being an integral part of the content to be incorporated by the general practitioner. Therefore, it is necessary to establish the essential VS competencies for medical practice in Brazil.

**Objectives:**

Define the most relevant themes in the VS for generalist medical training, developing a core curriculum (CC) for undergraduate medical courses in Brazil, and apply the matrix proposed in a pilot study with first-year resident doctors in internal medicine, gynecology and obstetrics, general surgery and family and community medicine.

**Methods:**

Construction and validation of content by ten professors of a CC proposal in VS for medical graduation, using the Delphi consensus method, with its subsequent application among residents, to evaluate it in the graduation experience of 35 graduates.

**Results:**

The instrument developed and validated in terms of content by the panel of teachers identified 17 of the 31 themes presented as essential, which were considered the CC of VS (anatomy, physiology, venous thromboembolism, diabetic foot and atherosclerosis). Peripheral arterial disease and extracranial cerebral ischemia were considered to have little or no development during medical graduation.

**Conclusions:**

The proposal and validation of a CC in VS, in the opinion of teachers, was a consensus and enabled the formulation of a concise and applicable research instrument. The qualification of the residents' experience on these topics served to understand which areas of teaching can be improved for primary medical care related to VS.

## INTRODUCTION

Medical school curricula should be developed within a new equilibrium between training of general practitioners and specialists, with considerable emphasis on prevention, focused on patients as people, and promoting concern for the health of individuals within their communities. They should also develop students’ capacity to critically analyze the effectiveness and cost of medical interventions, in order to prioritize the essential and avoid health care inequality.^1^

The core curriculum (CC) concept encapsulates a new way of approaching medical training.^2^ It is not simply a matter of summarizing content, but considers such summaries as a means to achieving a greater end: improvement of the medical degree as a whole, which involves rethinking objectives, content, teaching techniques, and assessment procedures.^3,4^

The objective of this study was to develop, validate, and administer questionnaires, followed by analysis of the data collected, preparatory to creation of a proposed VS CC for use in undergraduate medical degrees, and to administer this tool in a pilot study conducted with first-year resident physicians.

The Delphi consensus method was adopted for validation of the CC. This method for validation of the proposed instrument involves presenting a questionnaire to a panel of experts, who answer and evaluate it. After analysis of the data and incorporation of suggestions made by the panel, the questionnaire is sent back to the same experts. The process is repeated until there is agreement between at least 70% of the experts, at the end of which the consensus instrument is validated.

## METHODS

Faculty members who teach VS at Medical Schools in the Brazilian states of Paraná, Santa Catarina, Rio Grande do Sul, and São Paulo took part in the process to construct and validate the CC, constituting a convenience sample of professors. Five of these professors teach at public universities, four teach at private universities, and one teaches at both. The educational systems employed at the medical schools where these professors teach are as follows: six employ the traditional model, three use a problem-based learning model, and one uses a mixed model (traditional and modular model).

**Figure 1:**
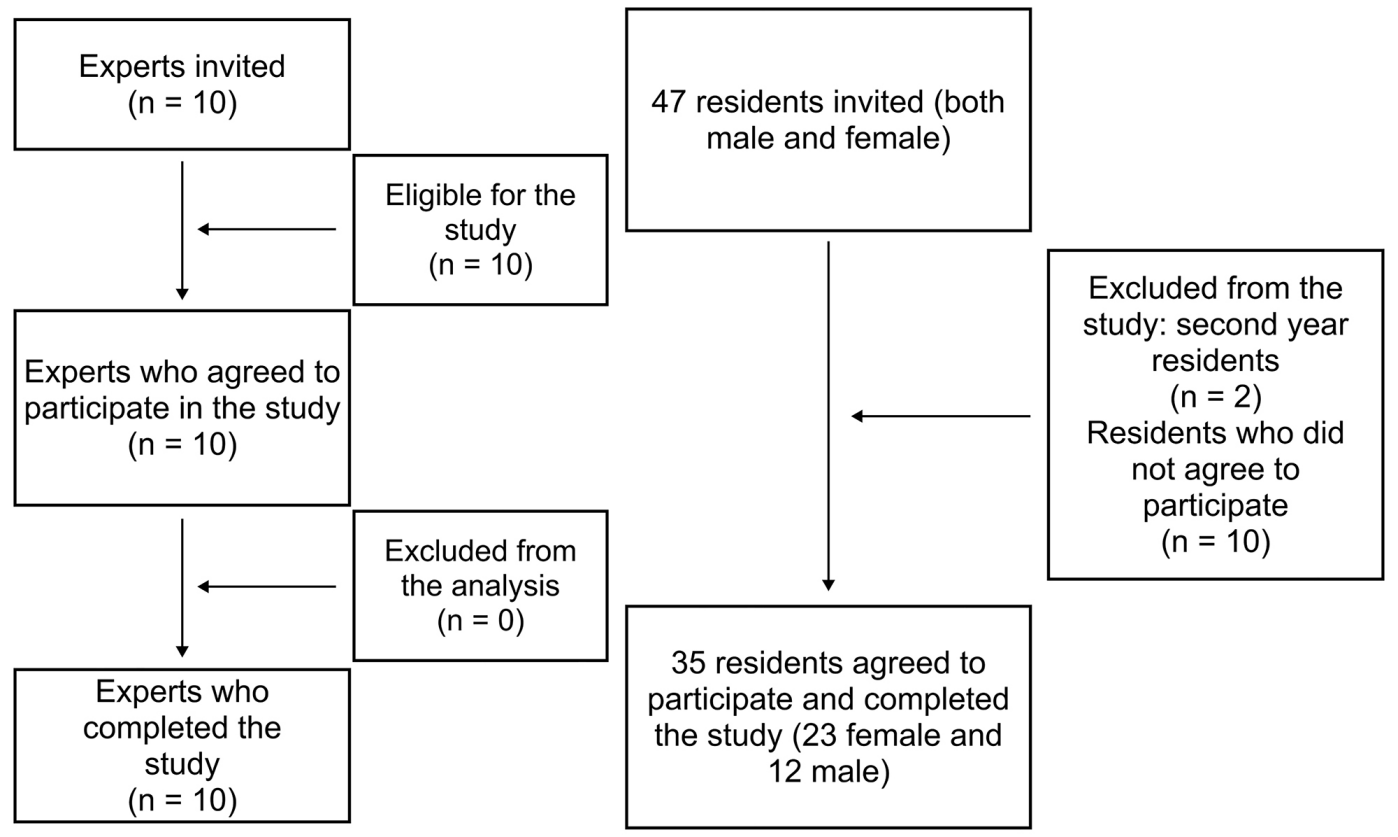
Image showing a flowchart illustrating inclusion, exclusion, and loss of study participants.

The pilot study administered the instrument to a small sample, used for cognitive interviews, which should comprise at least 10 to 30 participants. The actual sample comprised 35 participants (Figure 1), with the inclusion criteria defined as physicians on residencies in internal medicine, general surgery, gynecology and obstetrics, or family and community medicine. All of the participants were in their first year of medical residency, since having recently been through their training in VS, they would be able to comment on their memories of their experiences as medical undergraduates. Consequently, being a medical resident in later years was defined as an exclusion criterion. The study participants were selected by random convenience sampling.

All topics related to VS were compiled using sources from the specialty’s medical societies, both Brazilian and international, listing its theoretical and practical content. A research instrument was developed using this compilation of topics and then sent by electronic mail to 10 professors who teach VS and were asked to identify which topics were essential for undergraduate medical training.

An adapted version of the Fehring criteria^5^ (Table 1) was used, since it is widely used for content validation in studies in the health sciences. All of the professors enrolled scored more than 13 points according to these criteria and were thus considered qualified.

**Table 1:** Adapted Fehring criteria.^5^

**Adapted Fehring criteria**	**Score**
Master’s in Health Sciences	2 points
Doctorate in Health Sciences	4 points
Medical residency in Vascular Surgery	2 points
Participation in projects in the vascular area	2 points
Professional practice for more than 2 years	2 points
Experience in construction and administration of protocols	1 point
Published article on vascular surgery	2 points

**Figure 2:**
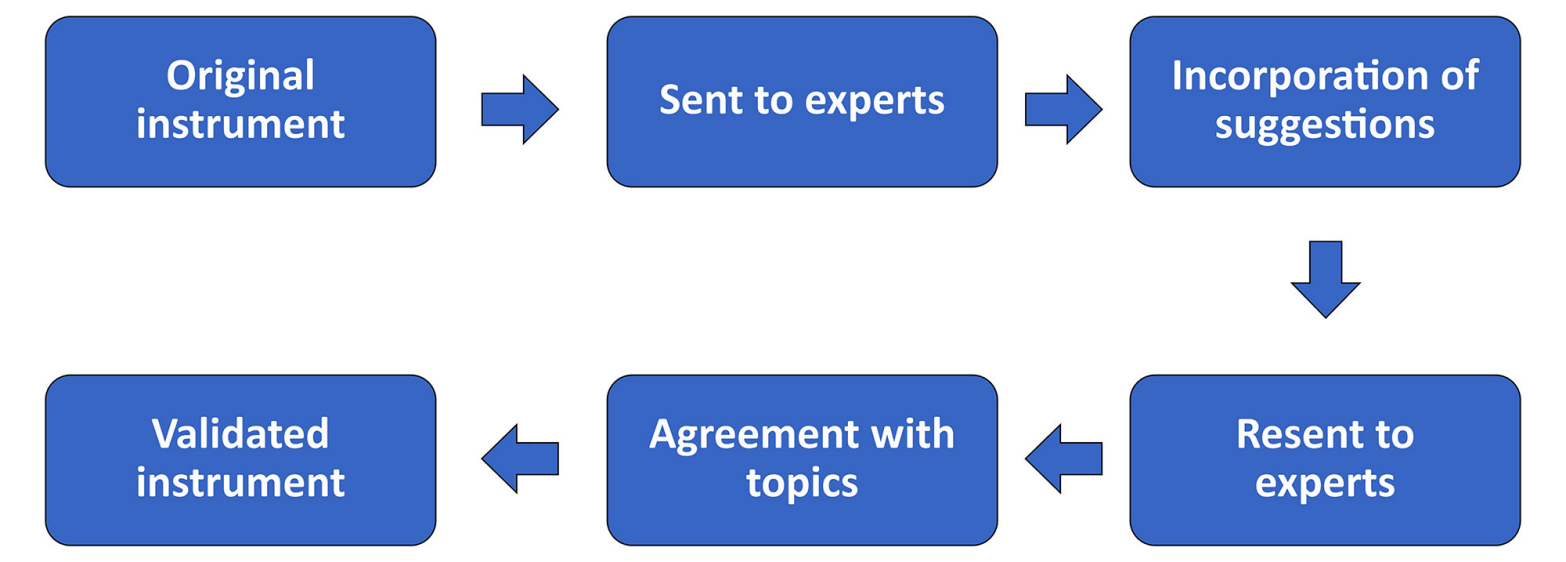
Flow diagram illustrating validation of the research instrument using the Delphi technique.

Each professor answered the questionnaire individually by electronic mail. Their responses were analyzed and recompiled and their suggestions were used to improve the instrument, which was then sent back to the expert panel members until there was at least 70% consensus for validation of the content (instrument B - Appendix A), as directed by the Delphi consensus technique (Figure 2).^6^ The validated content was used to construct the VS CC for undergraduate medical degree courses. Topics and skills identified by the expert panel as essential or highly important to knowledge of the VS specialty for general practitioners were included in the CC. Instrument B was then sent using Google^®^ forms to 35 first-year resident physicians training in internal medicine, general surgery, gynecology and obstetrics, or family and community medicine, selected by convenience sampling at hospitals and medical services in the four Brazilian states mentioned above, in order to assess their perceptions of their training in VS on their undergraduate courses.

The data collected using the questionnaires sent to the experts and to the resident physicians were organized and systematized digitally, using descriptive statistics, constructing simple and relative frequency tables (tabulation of data) and plots, in addition to cross-referencing the variables needed for general analysis and grouping data on each topic.

Each topic was surveyed using a Likert scale (Table 2) to classify the degree of agreement and the extent to which each topic was adequately covered, moderately covered, covered little, or not covered at all.^3^

**Table 2:** Likert scale.^3^

	ADEQUATELY COVERED
LIKERT ASSESSMENT	MODERATELY COVERED
SCALE	COVERED LITTLE
	NOT COVERED

Once the pilot study had been completed, the data tabulated and analyzed, and indices calculated, Cronbach’s alpha coefficient was calculated to determine the internal consistency of data reliability.^7^

This study was approved by the Human Research Ethics Committee at the Faculdades Pequeno Príncipe, under Ethics Appraisal Submission Certificate 55759822.6.0000.5580, opinion number 5.339.929.

## RESULTS

**Figure 3:**
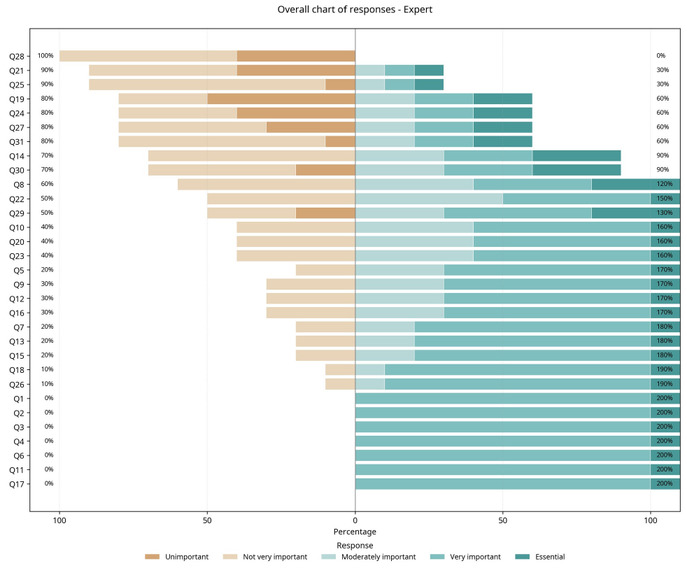
Experts’ ratings of importance of topics. Q = topics covered.

The expert panel rated each of the VS topics in order of importance using five categories: essential, highly important, moderately important, unimportant, or unnecessary. Topics rated as essential or highly important by the experts were selected for the CC (Table 3). Figure 3 illustrates the degree of importance of each of the topics, as rated by the expert panel of professors.

**Table 3:** Topics covered.

Q1 - Anatomy of the circulatory system
Q2 - Physiology of the circulatory system
Q3 - Vascular symptoms
Q4 - Current prevalence and importance of peripheral vascular diseases
Q5 - Hemostasis
Q6 - Superficial and deep venous thrombosis of the lower limbs and thrombophilias
Q7 - Pulmonary thromboembolism
Q8 - Thromboembolism in pregnancy and thrombophilias
Q9 - Varicose veins of the lower limbs
Q10 - Chronic venous insufficiency
Q11 - Atherosclerosis
Q12 - Aortoiliac atherosclerotic disease
Q13 - Femoropopliteal atherosclerotic disease and distal atherosclerotic disease
Q14 - Intestinal ischemia
Q15 - Cerebral ischemia of extracranial origin
Q16 - Aneurysms and arterial dissections
Q17 - Acute arterial occlusions
Q18 - Diabetic foot
Q19 - Lower limb amputations
Q20 - Vascular traumatisms
Q21 - Vasculitis, angiodysplasias and functional arteriopathies
Q22 - Lymphangitis, erysipelas and cellulitis
Q23 - Cutaneous ulcers
Q24 - Endovascular surgery
Q25 - Vascular access for hemodialysis
Q26 - Vascular procedures for the general practitioner
Q27 – Long-dwelling catheter implantation
Q28 - Thoracic outlet syndrome
Q29 - Vascular imaging

Topics rated as indispensable were selected for the CC, i.e. those rated by a majority (50% or more) of the experts as essential or highly important. Based on this definition, the topics considered essential were: anatomy of the circulatory system; physiology of the circulatory system; vascular symptoms; current prevalence and importance of peripheral vascular diseases; superficial and deep venous thrombosis of the lower limbs and thrombophilias; LL varicose veins; aneurysms and arterial dissections; acute arterial occlusions; diabetic foot; and vascular procedures for the general practitioner.

Topics the expert panel considered highly important, although not essential, were: atherosclerosis; hemostasis; pulmonary thromboembolism; aortoiliac atherosclerotic disease; femoropopliteal, and distal atherosclerotic disease; cerebral ischemia of extracranial origin; and lymphangitis, erysipelas, and cellulitis.

**Figure 4:**
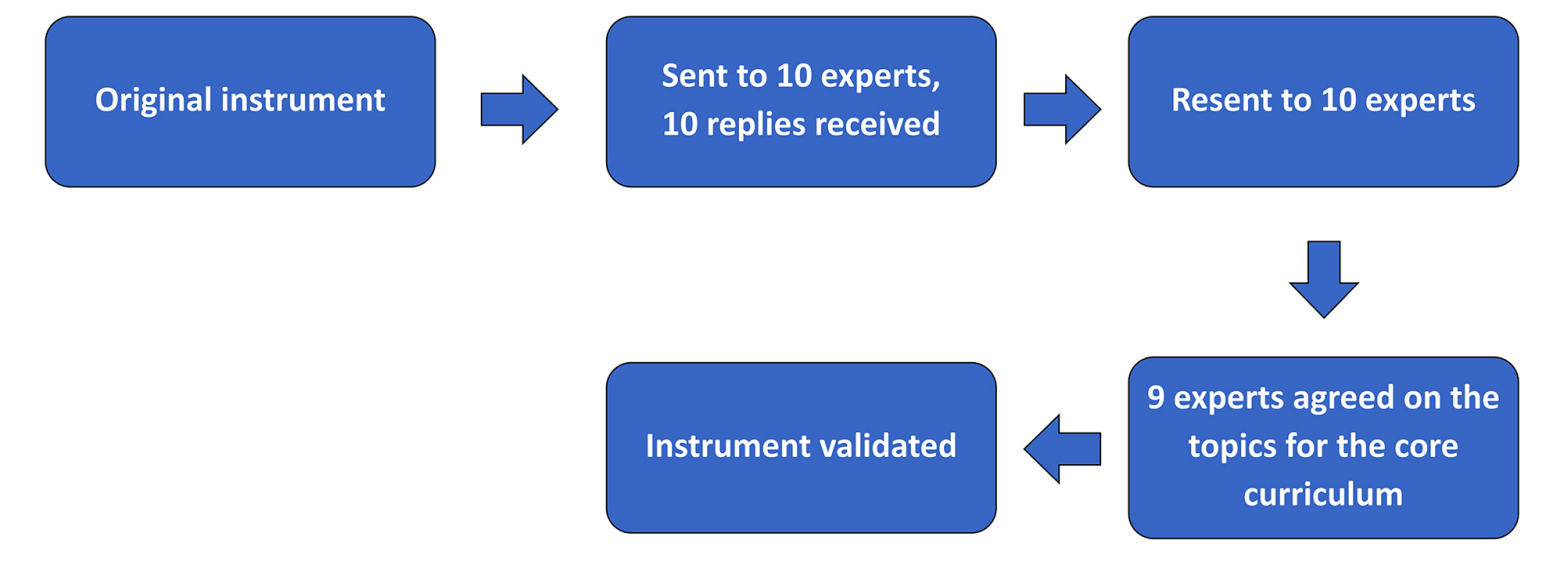
Flow diagram illustrating validation of the research instrument.

Analysis of the questionnaires resulted in a list of competencies containing 17 topics from the original total of 31 topics. These competencies, which had been reassessed by the expert panel using the Delphi consensus method, achieved agreement among more than 70% of the professors and were thus validated as the integrated VS CC instrument (Figure 4).

**Figure 5:**
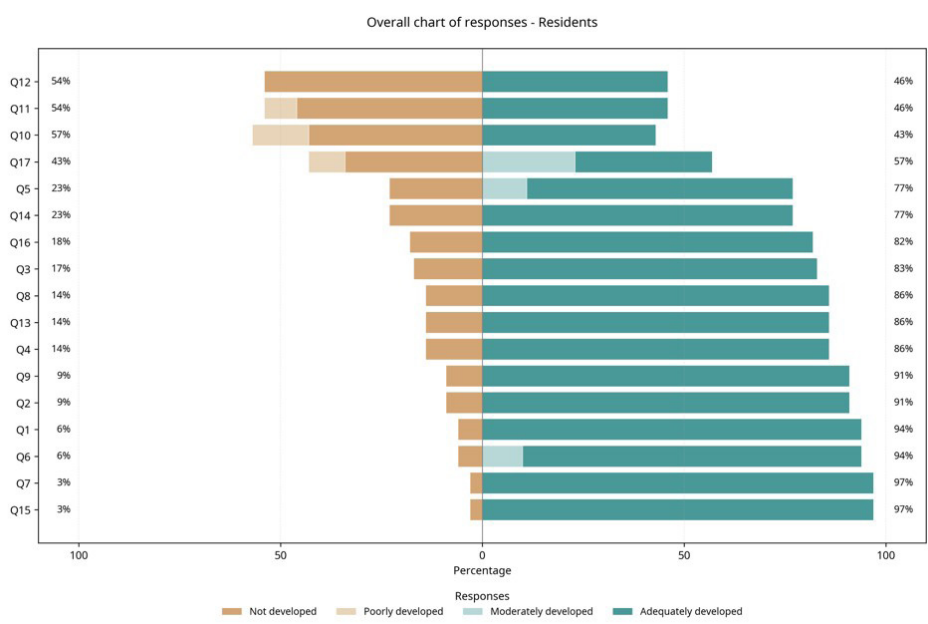
Degree of coverage of each topic in undergraduate medical courses and legend.

The validated instrument was then sent to the sample of resident physicians for them to assess the topics and skills listed. Each topic was rated on a Likert scale to classify the degree to which it was covered in their undergraduate courses, as adequately covered; moderately covered; covered little; or not covered at all. Twelve (34.3%) of the residents who agreed to take part and answered the questionnaire were male and 23 (65.7%) were female. With regard to their residency programs, three (8.57%) were on medical residencies in family medicine, 10 (28.56%) in internal medicine, three (8.57%) in gynecology and obstetrics, and 19 (54.3%) in general surgery (Figure 5).

**Figure 6:**
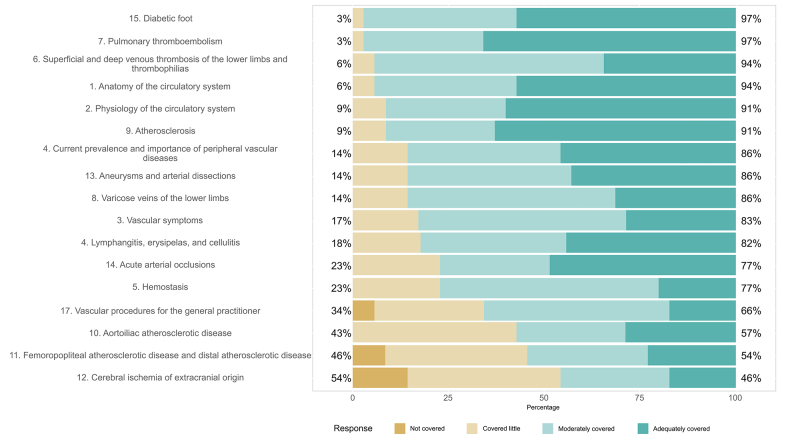
Results by topic covered.

Ten (28.57%) of the resident physicians who participated had graduated from public universities and 25 (71.43%) from private universities. The states in which they had graduated were as follows Paraná, 19 (54.29%); São Paulo, six (17.14%); Santa Catarina, seven (20.00%); and Rio Grande do Sul, three (8.57%). The mean time since graduation was 1.54 years. The overall results are illustrated in Figure 5. The results per topic covered are illustrated in Figure 6.

As shown in the data and graphs, basic topics considered essential or highly important, such as anatomy and physiology of the vascular system, were rated as adequately covered by the majority of the residents in the pilot study, with 57.1 and 60.0%, respectively. Other basic topics considered essential or highly important, such as vascular symptoms and current prevalence and importance of peripheral vascular diseases, were rated as adequately covered by 28.6 and 45.7% of the participants, respectively.

Among the topics involving basic knowledge of VS that were considered essential or highly important by the experts, hemostasis was rated as moderately covered in 57.1% of cases, while atherosclerosis was considered adequately covered in 62.9%.

Lymphangitis, erysipelas, and cellulitis; acute arterial occlusions; and aneurysms and arterial dissections were rated as adequately covered by 82, 77, and 86% of the residents, respectively.

More than 94% of the residents rated the topics diabetic foot; pulmonary thromboembolism; and superficial and deep venous thrombosis of the LL and thrombophilias as adequately or moderately covered. Cronbach’s alpha coefficient was 0.863.

## DISCUSSION

The objective of this study was to open a debate on the VS CC, which is very often considered secondary, in conjunction with a discussion at the management-administrative level or in relation to setting the teaching curriculum based on the curricula of more traditional institutions. In modern times, rethinking the CC from the perspective of the experience of students and provoking opportunities to readjust the skills included in curricula, focused on the perceptions of students and those delivering the educational activities provided, will be reflected in the future professional activities of a new generation capable of assimilating and executing the central competencies of all medical specialties that are essential for physicians’ daily practice. Therefore, the curricular guidelines for undergraduate courses in the health sciences also prioritize training professionals with a comprehensive view of clinical practice and a sense of social responsibility, capable not only of caring for patients, but also of empowering them and recognizing them as citizens and as unique individuals responsible for their own existence, aiming to fulfil the goal of, by the end of the course, training citizen- professionals who are both critical and reflective.^4^

In validation studies for diagnostic methods, interventions, or results, detailed description of the criteria for selection of experts is an essential step in guaranteeing the reliability of research findings and for their replication by other researchers.^4^

The pilot study collected sufficient data to identify topics and skills that are covered little and provided relevant context to identify deficiencies needing to be addressed. It was also possible to identify points of equilibrium between adequately covered topics, corroborating the consolidation of content validated in a list of competencies.^8^

Among topics directly related to vascular diseases and those dichotomized into anatomical areas, the subject of LL varicose veins was rated as moderately covered by 54.3% of residents, in dissonance with its high prevalence in the daily practice of general practitioners. In turn, atherosclerotic disease in aortoiliac, femoropopliteal, and distal areas, in conjunction with cerebral ischemia of extracranial origin, were identified as topics with the worst performance in the residents’ experience. These topics were considered to be covered little or not covered at all in terms of teaching, or were perceived in this manner by the residents when they were studying for their undergraduate degrees. This constitutes an incongruity, since the topic of atherosclerosis was rated as adequately covered, despite being the underlying pathophysiologic basis of these other topics that were covered less and which should have reflected a directly proportional degree of coverage.

The results of the pilot study with the resident physicians achieved a Cronbach’s Alpha of 0.863, demonstrating good consistency in terms of the reliability and internal consistency of the research instrument administered to the participants.

These data lead us to believe that important topics relating to teaching of VS are being covered in the institutions at which the resident physicians graduated. With regard to the degree of coverage of the subjects considered part of the proposed core curriculum, the results of the majority of the resident participants’ ratings indicated moderate or adequate coverage, classifying their education in each of the topics surveyed. A collective effort to change the current mindset, which considers certain specialties more or less worthy and important in the training of a general practitioner than others is a challenge, since this constitutes an obstacle to be overcome if a pertinent and virtuous education is to be achieved, to the benefit of the wellbeing of thousands of people.

In an article published in 2003, Panico et al.^9^ had already drawn attention to research conducted into the teaching of angiology and vascular surgery in Brazilian medical schools that showed that more than half of those graduating had received superficial and scattered education on vascular diseases, delivered by professors from other specialties.

This highlights the importance of defining a VS CC for undergraduate courses in medicine, which can serve as a foundation for the faculty members responsible for developing teaching plans and for teaching the subject, whether this be achieved by inclusion of dedicated modules, as in traditional curricula, or through integrated modules in which several different areas of knowledge act in conjunction. This would enable a focus on those topics considered essential and highly important, so that they could be adequately covered, guaranteeing effective learning during the undergraduate course, and so that they would be considered adequately covered in practically their entirety.^10^

## CONCLUSIONS

This study demonstrated the capacity to condense the list of competencies necessary to the training of general practitioners, defining a VS CC. In the professors’ opinions, the process to construct and validate this CC achieved consensus and supported development of a concise and applicable research instrument. Surveying the residents’ experience of VS topics served to understand and analyze which topics were covered sufficiently or could be altered or improved, in order to enhance VS-related primary health care.

## Appendix A Validated instrument sent to residents in the pilot study of application of the matrix.

Dear Colleague,

Please evaluate the following topics/contents and mark the option that best represents the degree of development of this topic in vascular surgery education during your medical school training, considering a core curriculum for the basic education of a general practitioner:

1. Anatomy of the circulatory system:

() Adequately covered

() Moderately covered

() Covered little

() Not covered

2. Physiology of the circulatory system:

() Adequately covered

() Moderately covered

() Covered little

() Not covered

3. Vascular symptoms:

() Adequately covered

() Moderately covered

() Covered little

() Not covered

4. Current prevalence and importance of peripheral vascular diseases:

() Adequately covered

() Moderately covered

() Covered little

() Not covered

5. Hemostasis:

() Adequately covered

() Moderately covered

() Covered little

() Not covered

6. Superficial and deep venous thrombosis of the lower limbs and thrombophilias;

() Adequately covered

() Moderately covered

() Covered little

() Not covered

7. Pulmonary thromboembolism:

() Adequately covered

() Moderately covered

() Covered little

() Not covered

8. Varicose veins of the lower limbs:

() Adequately covered

() Moderately covered

() Covered little

() Not covered

9. Atherosclerosis:

() Adequately covered

() Moderately covered

() Covered little

() Not covered

10. Aortoiliac atherosclerotic disease:

() Adequately covered

() Moderately covered

() Covered little

() Not covered

11. Femoropopliteal and distal atherosclerotic disease:

() Adequately covered

() Moderately covered

() Covered little

() Not covered

12. Cerebral ischemia of extracranial origin:

() Adequately covered

() Moderately covered

() Covered little

() Not covered

13. Aneurysms and arterial dissections:

() Adequately covered

() Moderately covered

() Covered little

() Not covered

14. Acute arterial occlusions:

() Adequately covered

() Moderately covered

() Covered little

() Not covered

15. Diabetic foot:

() Adequately covered

() Moderately covered

() Covered little

() Not covered

16. Lymphangitis, erysipelas and cellulitis:

() Adequately covered

() Moderately covered

() Covered little

() Not covered

17. Vascular procedures for the general practitioner (venous puncture; arterial puncture; venous dissection; arterial catheterization; complications and solutions):

() Adequately covered

() Moderately covered

() Covered little

() Not covered
