# Supplementary material for: Vascular surgery core curriculum for medical degrees in Brazil: a proposal
Source: J Vasc Bras. 2026 Feb 9;24:e20240039. doi: 10.1590/1677-5449.202400392 (PMC13042204; doi:10.1590/1677-5449.202400392)
Supplement: Translation

# Translation: *Core curriculum* de cirurgia vascular na graduação médica no Brasil: uma proposta

**DOI:** 10.1590/1677-5449.202400391

**Authors:** Alexandre Yoshiharu Shiomi, Juliano Mendes de Souza, Diancarlos Pereira de Andrade, Marcos Arêas Marques

**Affiliations:** 1 Clínica Angioprime, Curitiba, PR, Brasil.; 2 Universidade Federal do Paraná – UFPR, Curitiba, PR, Brasil.; 3 Faculdades Pequeno Príncipe, Curitiba, PR, Brasil.; 4 Universidade do Estado do Rio de Janeiro – UERJ, Hospital Universitário Pedro Ernesto, Rio de Janeiro, RJ, Brasil.

**Keywords:** currículo, educação baseada em competências, educação médica, doenças vasculares, procedimentos cirúrgicos vasculares, internato e residência

## Abstract

**Contexto:**

A cirurgia vascular (CV) é a área médica voltada ao estudo do sistema vascular, sendo parte integrante do conteúdo a ser incorporado pelo médico generalista. Assim, é necessário estabelecer as competências essenciais da CV para a prática médica no Brasil.

**Objetivos:**

Definir os temas de maior relevância na CV para a formação médica generalista, desenvolvendo um *core curriculum* para os cursos de graduação em Medicina no Brasil, e aplicar a matriz proposta em estudo piloto a médicos residentes do primeiro ano em clínica médica, ginecologia e obstetrícia, cirurgia geral e medicina da família e comunidade.

**Métodos:**

Construção e validação de conteúdo, por 10 professores, de uma proposta de *core curriculum* em CV para a graduação médica, utilizando o método de consenso de Delphi, com posterior aplicação entre residentes, a fim de avaliá-la na experiência da graduação de 35 egressos.

**Resultados:**

O instrumento desenvolvido e validado quanto ao conteúdo, pelo painel de professores, identificou 17 dos 31 temas apresentados como essenciais, os quais foram considerados o *core curriculum* de CV (anatomia, fisiologia, tromboembolismo venoso, pé diabético e aterosclerose). A doença arterial periférica e a isquemia cerebral extracraniana foram assuntos considerados pouco ou nada desenvolvidos durante a graduação médica.

**Conclusões:**

A proposta e a validação de um *core curriculum* em CV, na opinião de professores, alcançaram consenso e possibilitaram a formulação de um instrumento de pesquisa conciso e aplicável. A qualificação da experiência nesses temas, pelos residentes, serviu para entender quais áreas de ensino podem ser melhoradas para a assistência médica primária relacionada à CV.

## INTRODUÇÃO

O currículo médico deve ser construído dentro de um novo equilíbrio na produção de generalistas e especialistas, com grande ênfase na prevenção e foco no paciente como pessoa, ampliando a preocupação com a saúde do indivíduo em sua comunidade. Além disso, deve ser desenvolvida a habilidade para avaliar criticamente a efetividade e o custo de intervenções médicas, a fim de priorizar o que é essencial e manter uma equidade no cuidado da saúde^1^.

O *core curriculum* (CC) traduz uma nova maneira de encarar o ensino médico^2^. Não se trata apenas de resumir conteúdos, mas, sim, de considerar esse resumo como um meio para atingir um fim maior: o aprimoramento da graduação médica como um todo, o que envolve repensar objetivos, conteúdos, técnicas instrucionais e procedimentos avaliativos^3,4^.

O objetivo deste estudo foi o desenvolvimento, a validação e a submissão de questionários, com posterior análise das informações coletadas, bem como a criação de uma proposta de um CC de CV para a graduação em Medicina e a aplicação dessa ferramenta em um estudo piloto entre médicos residentes do primeiro ano.

Para a validação do CC, o método de consenso de Delphi foi escolhido, o qual consiste na validação do instrumento proposto, onde um questionário é submetido a um painel de *experts*, respondido e avaliado. Após a análise dos dados, com o adendo das sugestões realizadas por esse painel, o questionário é reenviado aos mesmos especialistas. O processo ocorre até que haja uma concordância de mais de 70% entre os especialistas, validando o instrumento consensual ao seu final.

## MÉTODOS

Para a construção e a validação da matriz curricular, participaram da pesquisa docentes de CV de cursos de Medicina dos estados do Paraná, Santa Catarina, Rio Grande do Sul e São Paulo, os quais compuseram o grupo de professores por uma amostra de conveniência. Desses, cinco professores são docentes de universidades públicas, quatro de universidades privadas e um leciona em ambas. Entre os sistemas educacionais onde os professores atuam, seis adotam o modelo tradicional, três o modelo de aprendizagem baseada em problemas, e um o modelo misto (tradicional e modelo modular).

A aplicação do instrumento no estudo piloto foi condicionada a uma amostra de tamanho pequeno, utilizada para entrevistas cognitivas, nas quais devem ser envolvidos pelo menos 10 a 30 participantes. Assim, foram incluídos 35 participantes (Figura 1), com critérios de inclusão populacional definidos para médicos residentes de clínica médica, cirurgia geral, ginecologia e obstetrícia e medicina de família e comunidade. Todos os participantes eram do primeiro ano de residência médica, por terem passado recentemente pelo aprendizado em CV, trazendo à tona as lembranças de suas experiências como discentes da graduação médica. Consequentemente, foi definido como critério de exclusão ser médico residente de anos subsequentes. Os participantes do estudo foram selecionados por amostra de conveniência e aleatória.

**Figura 1:** Imagem demonstrando o *flowchart*, com inclusão, exclusão e perda dos participantes do estudo.

Foram compilados todos os temas de CV a partir de fontes relacionadas às sociedades médicas da especialidade, nacionais e internacionais, elencando-se seus conteúdos teóricos e práticos. Um instrumento de pesquisa foi desenvolvido com esse compilado de temas e, em seguida, encaminhado, por meio de correio eletrônico, para 10 professores da área de ensino em CV, para que pudessem identificar os temas essenciais para o ensino da área na graduação médica.

Optou-se por adotar os critérios de Fehring adaptados (Tabela 1)^5^, por serem amplamente utilizados nas pesquisas relacionadas à área da saúde para validação de conteúdo. Todos os professores selecionados somaram mais de 13 pontos, segundo os critérios supracitados, sendo considerados qualificados.

**Tabela 1:** Critérios de Fehring adaptados^5^.

**Critérios de Fehring adaptados**	**Pontuação**
Mestrado em Ciências da Saúde	2 pontos
Doutorado em Ciências da Saúde	4 pontos
Residência Médica em Cirurgia Vascular	2 pontos
Participação em projetos na área vascular	2 pontos
Atuação profissional por mais de 2 anos	2 pontos
Experiência em construção e aplicação de protocolos	1 ponto
Artigo publicado em Cirurgia Vascular	2 pontos

Cada professor respondeu individualmente ao questionário por meio de correio eletrônico, de forma individual. Suas respostas foram analisadas e compiladas novamente, e suas sugestões consideradas, a fim de aperfeiçoar o instrumento, o qual foi reencaminhado, por meio de correio eletrônico, para os especialistas participantes do painel, até que houvesse consenso de 70% para validação do conteúdo (instrumento B - Apêndice A), conforme a técnica de consenso de Delphi (Figura 2)^6^. Após a validação do conteúdo, foi criado o CC de CV para a graduação em Medicina. Foram considerados como componentes integrantes do CC os conteúdos e habilidades apontados pelo painel de especialistas como essenciais ou muito importantes ao conhecimento da especialidade de CV para os médicos generalistas. O instrumento B foi encaminhado por correio eletrônico, através de formulário do Google^®^, para 35 médicos residentes do primeiro ano de clínica médica, cirurgia geral, ginecologia e obstetrícia e medicina de família e comunidade, selecionados por amostra de conveniência em hospitais e serviços médicos dos quatro estados da federação mencionados, a fim de avaliar a percepção do aprendizado em CV durante a graduação.

**Figura 2:** Fluxograma de validação do instrumento de pesquisa pela técnica de Delphi.

Os dados coletados dos questionários enviados aos professores e aos médicos residentes foram organizados e sistematizados em meio digital, utilizando estatística descritiva, com a construção de tabelas de frequências simples e relativas (tabulação dos dados) e gráficos, bem como cruzamentos de variáveis necessárias para a análise geral e o agrupamento das informações do tema.

Cada um dos conteúdos foi acompanhado de uma escala de Likert (Tabela 2) para classificar o grau de concordância e desenvolvimento dos temas como adequadamente desenvolvido, medianamente desenvolvido, pouco desenvolvido ou não desenvolvido (indiferente)^3^.

**Tabela 2:** Escala de Likert^3^.

**ESCALA**	**ADEQUADAMENTE DESENVOLVIDO**
DE LIKERT	MEDIANAMENTE DESENVOLVIDO
PARA AVALIAÇÃO	POUCO DESENVOLVIDO
	NÃO DESENVOLVIDO

Uma vez aplicados o estudo piloto, a análise dos dados com suas devidas tabulações e os cálculos de índices, foi realizado o coeficiente alfa de Cronbach para avaliar a consistência interna de confiabilidade dos dados^7^.

Este estudo foi aprovado pelo Comitê de Ética em Pesquisa em Seres Humanos da Faculdades Pequeno Príncipe, sob o registro do Certificado de Apresentação de Apreciação Ética 55759822.6.0000.5580, parecer nº 5.339.929.

## RESULTADOS

O grupo de professores julgou cada um dos temas de interesse da CV em uma das cinco categorias, em ordem de importância: essencial, muito importante, medianamente importante e pouco importante ou desnecessário. Os temas considerados para o CC foram os avaliados pelos especialistas como essenciais ou muito importantes (Tabela 3). O grau de importância dos temas, conforme julgado pelo grupo de professores, encontra-se na Figura 3.

**Tabela 3:** Temas abordados.

Q1 - Anatomia do sistema circulatório
Q2 - Fisiologia do sistema circulatório
Q3 - Semiologia vascular
Q4 - Prevalência e importância das doenças vasculares periféricas na atualidade
Q5 - Hemostasia
Q6 - Trombose venosa superficial e profunda dos membros inferiores e trombofilias
Q7 - Tromboembolismo pulmonar
Q8 - Tromboembolismo na gestação e trombofilias
Q9 - Varizes dos membros inferiores
Q10 - Insuficiência venosa crônica
Q11 - Aterosclerose
Q12 - Doença aterosclerótica aortoilíaca
Q13 - Doença aterosclerótica femoropoplítea e doença aterosclerótica distal
Q14 - Isquemia intestinal
Q15 - Isquemia cerebral de origem extracraniana
Q16 - Aneurismas e dissecções arteriais
Q17 - Oclusões arteriais agudas
Q18 - Pé diabético
Q19 - Amputações de membros inferiores
Q20 - Traumatismos vasculares
Q21 - Vasculites, angiodisplasias e arteriopatias funcionais
Q22 - Linfangite, erisipela e celulite
Q23 - Úlceras cutâneas
Q24 - Cirurgia endovascular
Q25 - Acesso vascular para hemodiálise
Q26 - Procedimentos vasculares para o médico generalista
Q27 - Implantação de cateter de longa permanência
Q28 - Síndrome do desfiladeiro torácico
Q29 - Imagem vascular

**Figura 3:** Grau de importância dos temas pelos professores. Q = tema abordado.

Para o desenvolvimento do CC, foram considerados temas indispensáveis, ou seja, aqueles caracterizados pela maioria (50% ou mais) dos professores como essenciais ou muito importantes. Com base nessa definição, os temas considerados essenciais foram: anatomia, fisiologia do sistema circulatório, semiologia vascular, prevalência e importância das doenças vasculares periféricas na atualidade, trombose venosa superficial e profunda dos membros inferiores (MMII), trombofilias, varizes dos MMII, aneurismas e dissecções arteriais, oclusões arteriais agudas, pé diabético e procedimentos vasculares para o médico generalista.

Os temas considerados muito importantes, porém, não essenciais pelo grupo de professores, foram: aterosclerose, hemostasia, tromboembolismo pulmonar, doença aterosclerótica aortoilíaca, femoropoplítea e distal, isquemia cerebral de origem extracraniana, e linfangite, erisipela e celulite.

A partir da análise dos questionários, do total de 31 temas, obteve-se a composição de uma matriz de competências com 17 temas. Essas competências, reavaliadas pelo painel de especialistas por meio do método de consenso de Delphi, apresentaram concordância de mais de 70% entre os professores, sendo, assim, validadas como instrumento do CC integrado em CV (Figura 4).

**Figura 4:** Fluxograma de validação do instrumento de pesquisa.

O instrumento validado foi encaminhado aos médicos residentes para avaliação dos conteúdos e habilidades. Cada um dos conteúdos foi acompanhado de uma escala de Likert para classificar o grau de desenvolvimento durante a graduação como adequadamente desenvolvido, medianamente desenvolvido, pouco desenvolvido ou não desenvolvido. Entre os residentes que concordaram em participar e responderam ao questionário, 12 (34,3%) são do sexo masculino e 23 (65,7%) do sexo feminino. Em relação ao programa de residência que frequentam, três (8,57%) fazem residência médica em medicina de família, 10 (28,56%) em clínica médica, três (8,57%) em ginecologia e obstetrícia, e 19 (54,3%) em cirurgia geral (Figura 5).

**Figura 5:** Grau de desenvolvimento dos temas durante a graduação e legendas.

Entre os médicos residentes participantes, 10 (28,57%) são graduados em faculdades públicas e 25 (71,43%) em faculdades privadas. Com relação ao estado onde se formaram, 19 (54,29%) são do Paraná, seis (17,14%) de São Paulo, sete (20,00%) de Santa Catarina e três (8,57%) do Rio Grande do Sul. A média de tempo de formado ficou registrada em 1,54 anos. Os resultados encontrados são apresentados na Figura 5. Os resultados por tema abordado estão representados na Figura 6.

**Figura 6:** Resultados por tema abordado.

Conforme os dados e gráficos apresentados nos resultados da pesquisa, temas básicos determinados como essenciais ou muito importantes, como a anatomia e fisiologia do sistema vascular, foram considerados adequadamente desenvolvidos pela maioria dos residentes no estudo piloto, com 57,1 e 60,0%, respectivamente. Outros temas básicos considerados como essenciais ou muito importantes, como a semiologia vascular e a prevalência e importância das doenças vasculares periféricas na atualidade, foram tratados como adequadamente desenvolvidos por 28,6 e 45,7% dos participantes, respectivamente.

Entre os temas de ciência básica da CV considerados essenciais ou muito importantes, a hemostasia foi classificada como medianamente desenvolvida em 57,1% dos casos, enquanto a aterosclerose foi considerada adequadamente desenvolvida em 62,9%.

Linfangite, erisipela e celulite, oclusões arteriais agudas, aneurismas e dissecções arteriais foram considerados adequadamente desenvolvidos por 82, 77 e 86% dos residentes, respectivamente.

Segundo os residentes, os temas pé diabético, tromboembolismo pulmonar, trombose venosa superficial e profunda dos MMII e trombofilias apresentaram desenvolvimento adequado ou mediano em mais de 94% das respostas. O coeficiente alfa de Cronbach obtido foi de 0,863.

## DISCUSSÃO

O objetivo deste estudo foi trazer à tona a reflexão acerca do CC em CV, muitas vezes considerado de caráter secundário, associado a uma discussão em nível gestor-administrativo ou a uma programação de grade curricular docente baseada em currículos de instituições mais tradicionais. Modernamente, repensar o CC a partir da experiência discente, promovendo oportunidades de readequação das matrizes de competências curriculares, centradas na percepção dos estudantes e dos executores das ações educacionais promovidas, refletirá nos atos profissionais futuros de uma nova geração capaz de assimilar e desempenhar competências centrais de todas as especialidades médicas fundamentais para o trabalho cotidiano do médico. Assim, ﻿as diretrizes curriculares dos cursos de graduação na área da saúde priorizam, também, a formação de um profissional com uma visão ampliada da clínica e um senso de responsabilidade social, capaz não apenas de atender o paciente, mas de torná-lo e reconhecê-lo como cidadão, sujeito único e responsável por sua existência, visando atingir, ao final do curso, a meta de formação do profissional-cidadão, crítico e reflexivo^4^.

Nos estudos de validação de diagnósticos, intervenções ou resultados, a descrição detalhada dos critérios de seleção dos professores configura um passo fundamental para garantir a confiabilidade dos achados da pesquisa, bem como a replicação destes por outros pesquisadores^4^.

Na aplicação piloto, foram coletados dados suficientes para identificar conteúdos e habilidades pouco desenvolvidos, bem como um contexto de relevância acerca das deficiências a serem sanadas. Também foi possível identificar pontos de equilíbrio entre conteúdos adequadamente desenvolvidos, que corroboram o fortalecimento do conteúdo validado em uma matriz de competências^8^.

Dos temas relacionados diretamente às doenças vasculares, bem como aqueles dicotomizados em segmentos por território de abordagem, o tema varizes de MMII foi classificado como medianamente desenvolvido em 54,3%, em dissonância com sua alta prevalência no cotidiano de um médico generalista. A doença aterosclerótica aortoilíaca, do território femoropoplíteo e distal, combinada à isquemia cerebral de origem extracraniana, foi apontada como tendo o pior desempenho na experiência dos residentes. Esses temas foram considerados pouco ou não desenvolvidos do ponto de vista de ensino, ou percebidos dessa forma pelos residentes em sua época de graduação. Esse achado revela uma incoerência, já que o tema relacionado à aterosclerose foi considerado adequadamente desenvolvido, embora seja a base fisiopatológica para os demais temas menos desenvolvidos, o que deveria refletir um grau de desenvolvimento diretamente proporcional.

Na aplicação do estudo piloto aos médicos residentes, alcançou-se o valor de 0,863 do alfa de Cronbach, demonstrando uma boa consistência no que diz respeito à confiabilidade e à consistência interna do instrumento de pesquisa aplicado aos participantes.

Esses dados levam a acreditar que temas importantes relativos ao ensino da CV estão sendo trabalhados nas instituições das quais os médicos residentes da pesquisa são egressos. Os resultados da avaliação feita pelos participantes, em relação ao grau de desenvolvimento dos assuntos considerados para a matriz curricular desenvolvida, apresentam, em sua maioria, desenvolvimento mediano ou adequado, qualificando o aprendizado dos pesquisados em relação a cada tema. Um esforço conjunto para modificar a mentalidade presente, que considera determinadas especialidades mais ou menos nobres que outras e não importantes para a formação de um médico generalista, constitui um desafio e uma barreira a ser rompida, a fim de que floresça um ensino pertinente e virtuoso, em prol do bem-estar de milhares de pessoas.

Panico et al.^9^, em artigo publicado em 2003, já chamavam a atenção para pesquisa realizada sobre o ensino de angiologia e cirurgia vascular em faculdades de Medicina no Brasil, que constatou que mais da metade dos graduandos recebia ensinamento superficial e pulverizado sobre as doenças vasculares, ministrado por docentes de outras especialidades.

Este fato aponta a importância de se definir um CC de CV para a graduação em Medicina, que possa servir de base para os docentes responsáveis pela elaboração do plano de aulas e pelo ensino da disciplina, seja por meio de inserção dentro de disciplinas próprias, como no currículo tradicional, seja em disciplinas integradas, com várias áreas do conhecimento atuando em conjunto. Dessa forma, torna-se possível enfocar em temas considerados essenciais e muito importantes, para que sejam trabalhados de maneira adequada, garantindo aprendizado efetivo durante a graduação e sendo considerados adequadamente desenvolvidos em praticamente sua totalidade^10^.

## CONCLUSÃO

Foi demonstrada a capacidade de condensar a matriz de competências necessárias para a formação de um médico generalista, estabelecendo um CC em CV. A proposta e a validação desse CC, na opinião dos professores, demonstraram consenso e possibilitaram a formulação de um instrumento de pesquisa conciso e aplicável. A qualificação da experiência nos temas de CV, pelos residentes, permitiu compreender e analisar quais conteúdos foram suficientemente abordados, podendo ser alterados ou melhorados, de modo a melhorar a assistência médica primária relacionada à CV.

## Apêndice A Instrumento validado enviado aos residentes no estudo piloto de aplicação da matriz.

Prezado Colega,

Favor avaliar os temas/conteúdos a seguir e assinalar a alternativa que compreende o grau de desenvolvimento deste tema no ensino da cirurgia vascular na sua graduação do curso de medicina, considerando um *core curriculum* para a formação básica de um

1. Anatomia do sistema circulatório:

() Adequadamente desenvolvida

() Medianamente desenvolvida

() Pouco desenvolvida

() Não desenvolvida

2. Fisiologia do sistema circulatório:

() Adequadamente desenvolvida

() Medianamente desenvolvida

() Pouco desenvolvida

() Não desenvolvida

3. Semiologia vascular:

() Adequadamente desenvolvida

() Medianamente desenvolvida

() Pouco desenvolvida

() Não desenvolvida

4. Prevalência e importância das doenças vasculares periféricas na atualidade:

() Adequadamente desenvolvida

() Medianamente desenvolvida

() Pouco desenvolvida

() Não desenvolvida

5. Hemostasia:

() Adequadamente desenvolvida

() Medianamente desenvolvida

() Pouco desenvolvida

() Não desenvolvida

6. Trombose venosa superficial e profunda dos membros inferiores e trombofilias:

() Adequadamente desenvolvida

() Medianamente desenvolvida

() Pouco desenvolvida

() Não desenvolvida

7. Tromboembolismo Pulmonar:

() Adequadamente desenvolvida

() Medianamente desenvolvida

() Pouco desenvolvida

() Não desenvolvida

8. Varizes dos membros inferiores:

() Adequadamente desenvolvida

() Medianamente desenvolvida

() Pouco desenvolvida

() Não desenvolvida

9. Aterosclerose:

() Adequadamente desenvolvida

() Medianamente desenvolvida

() Pouco desenvolvida

() Não desenvolvida

10. Doença aterosclerótica aorto-ilíaca:

() Adequadamente desenvolvida

() Medianamente desenvolvida

() Pouco desenvolvida

() Não desenvolvida

11. Doença aterosclerótica femoro-poplítea e doença aterosclerótica distal:

() Adequadamente desenvolvida

() Medianamente desenvolvida

() Pouco desenvolvida

() Não desenvolvida

12. Isquemia cerebral de origem extracraniana:

() Adequadamente desenvolvida

() Medianamente desenvolvida

() Pouco desenvolvida

() Não desenvolvida

13. Aneurismas e dissecções arteriais:

() Adequadamente desenvolvida

() Medianamente desenvolvida

() Pouco desenvolvida

() Não desenvolvida

14. Oclusões arteriais agudas:

() Adequadamente desenvolvida

() Medianamente desenvolvida

() Pouco desenvolvida

() Não desenvolvida

15. Pé diabético:

() Adequadamente desenvolvida

() Medianamente desenvolvida

() Pouco desenvolvida

() Não desenvolvida

16. Linfangite, erisipela e celulite:

() Adequadamente desenvolvida

() Medianamente desenvolvida

() Pouco desenvolvida

() Não desenvolvida

17. Procedimentos vasculares para o médico generalista (punção venosa; punção arterial; dissecção venosa; cateterismo arterial; complicações e soluções):

() Adequadamente desenvolvida

() Medianamente desenvolvida

() Pouco desenvolvida

() Não desenvolvida
